# The Physiology of the Domestic Animals

**Published:** 1889-09

**Authors:** 


**The Physiology of the Domestic Animals. A text-book for veterinary and medical students and practitioners.** By ROBERT MEADE SMITH, A. M., M. D., Professor of Comparative Physiology in the University of Pennsylvania; Fellow of the College of Physicians and Academy of the Natural Sciences, Philadelphia; of the American Physiological Society; of the American Society of Naturalists, Associe etranger de la Société Francaise d'Hygiene, etc. With over 400 illustrations. 8vo, pp. 675. Philadelphia and London: F. A. Davis. 1889. Cloth, $6; sheep, $6.75 net.

It is not often that the medical profession has the opportunity of reading a new book upon a new subject, and doubtless English-speaking physicians will feel grateful to Prof. Smith for his admirable and pioneer work in a branch of medical science upon which a great amount of ignorance prevails. We have many times noted most peculiar, even ridiculous, notions concerning the anatomy and physiology of such animals as the horse, dog, and cat, and this among medical men. When, however, we reflect upon the limitations of city life in the study of the animal kingdom, and the lack of any reliable literature upon the subject, it is not such a wonder that the farmer, with larger opportunities for observation and tradition to teach him, should surpass even the doctor in this line of knowledge.

Under the heading General Physiology, the author first considers cells, discussing, in turn, their anatomy, physics, and chemistry in a most complete and satisfactory manner. The importance which the author attaches to a comprehensive knowledge of cells, appears throughout the book. Beginning with the simplest forms of various physiological processes, he traces their development up through the higher grades of animal life until he has reached the most complex forms of the mammalian kingdom. This mode of discussing the subject is very valuable to the student, and is one we should like to see more frequently employed by authors.

The second division (Special Physiology) comprises the greater part of the book, and contains some chapters on the feeding and nutrition of animals, and the physiology of movement, which are especially good.

The last portion of the work is devoted to the reproductive functions, and contains much valuable information upon a portion of animal physiology concerning which many are ignorant.

The cuts, some of them colored, are numerous and good. They are taken from standard works upon the same or allied subjects, credit being given in each instance. The book is a most valuable one in every way, and will be consulted largely by veterinary and medical students and practitioners. J. P.

